# Antibody elution with 2-me/SDS solution: Uses for multi-layer immunohistochemical analysis of wholemount preparations of human colonic myenteric plexus

**DOI:** 10.1016/j.heliyon.2024.e26522

**Published:** 2024-02-21

**Authors:** Adam Humenick, M.E. Johnson, B.N. Chen, M. Wee, D.A. Wattchow, M. Costa, P.G. Dinning, S.J.H. Brookes

**Affiliations:** a Human Physiology, College of Medicine and Public Health, Flinders University, Bedford Park, South Australia, 5042, Australia; b Department of Surgery, Flinders Medical Centre, Bedford Park, SA 5042, Australia

**Keywords:** Fluorescent antibody technique, Indirect, Protein denaturation, Lipofuscin, Myenteric plexus, Enteric nervous system

## Abstract

Indirect immunofluorescence is usually restricted to 3-5 markers per preparation, limiting analysis of coexistence. A solution containing 2-mercaptoethanol and sodium dodecyl sulfate (2-ME/SDS) can elute indirect immunofluorescence labelling (i.e. primary antisera followed by fluorophore-conjugated secondary antisera) and has been used for sequential staining of sections. The aim of this study was to test whether 2-ME/SDS is effective for eluting indirect immunofluorescent staining (with primary antisera visualised by fluorophore-coupled secondary antisera) in wholemount preparations. We also analysed how 2-ME/SDS may work and used this understanding to devise additional uses for immunofluorescence in the nervous system. 2-ME/SDS appears to denature unfixed proteins (including antisera used as reagents) but has much less effect on antigenicity of formaldehyde-fixed epitopes. Moieties linked by strong biotin-streptavidin bonds are highly resistant to elution by 2-ME/SDS. Two primary antisera raised in the same species can be applied without spurious cross-reactivity, if a specific order of labelling is followed. The first primary antiserum is followed by a biotinylated secondary, then a tertiary of fluorophore-conjugated streptavidin. The preparation is then exposed to 2-ME/SDS, which has minimal impact on labelling by the first primary/secondary/tertiary combination. However, when this is followed by a second primary antiserum (raised in the same species), followed by a fluorophore-conjugated secondary antiserum, the intervening 2-ME/SDS exposure prevents cross-reactivity between primary and secondary antisera of the two layers. A third property of 2-ME/SDS is that it reduces lipofuscin autofluorescence, although it also raises background fluorescence and strongly enhances autofluorescence of erythrocytes. In summary, 2-ME/SDS is easy to use, cost-effective and does not require modified primary antisera. It can be used as the basis of a multi-layer immunohistochemistry protocol and allows 2 primary antisera raised in the same species to be used together.

## Highlights

• 2-ME/SDS solution elutes indirect immunofluorescence labelling from thick wholemount preparations.
• It does not elute biotinylated secondary antisera binding streptavidin-fluorophore.
• 2-ME/SDS can reduce cross reactivity between antisera raised in the same species.
• 2-ME/SDS reduces autofluorescence of lipofuscins.

## 1 Introduction

Neurons have a wide range of morphological, electrophysiological and neurochemical properties, as well as distinctive projections and connections. To analyse how circuits work, it is useful to divide neurons into classes that share similar properties [1]. One way to do this is to use immunohistochemical labelling to localise cell markers. Notwithstanding the power of single cell transcriptome analysis [2], the “chemical coding” of a neuron (i.e. the combination of neuronal markers that it expresses), has been extensively used as a basis for characterising neurons in both central [3] and peripheral nervous circuits [4]. Typically, this is achieved using multiple immunofluorescence labelling to assess co-existence of markers. With Raman dye imaging it is possible to analyse up to 11 markers simultaneously [5] but without specialised microscopy systems, indirect immunofluorescence is restricted to 4-5 markers, the so-called “colour barrier" which limits the number of non-overlapping fluorescence channels [5]. There is also a need to have primary antisera raised in different host species [6].

Usually, 3-4 markers are insufficient to assign all neurons to classes. This makes it necessary to study multiple preparations, with overlapping combinations of markers, then combine the results [7]. In mathematical terms, errors sum with every combination and quantitation of classes therefore becomes less and less precise. Several methods have been developed to overcome this limitation. Use of monovalent F (ab) secondary antisera allow two or more primary antisera raised in the same species to be applied successively. This requires careful optimisation of concentrations and incubations [8]. Multiplexed immunofluorescence can be achieved using primary antisera conjugated directly to fluorescent cyanine dyes. These fluorophores can be inactivated with mild solutions that do not damage epitopes in the tissue. This makes it possible to sequentially label more than 60 markers in the same cells [9]. However, this method requires fluorophore-conjugated primary antisera for every marker of interest. “Multi-epitope ligand cartography/toponome imaging system” (MELC/TIS) uses directly labelled probes applied sequentially, separated by bouts of photo-bleaching. Using a partially automated system, up to 100 proteins have been localised in a single cell [. This method also depends on the availability of a library of direct dye-conjugated primary antisera [10]. Methods for multiplexing have been reviewed recently [11]).

Another approach is to elute primary and secondary antisera from tissue using modified stripping buffers, similar to those used in Western blots. They use heat, low or high pH solutions, denaturing solutes (urea, glycine or guanidine hydrochloride) or reducing agents (2-mercaptoethanol or dithiothreitol) to displace the antibodies. Elution has been successfully used in tissue sections 3-4 μm thick and in tissue microarrays. We recently applied the protocol of Gendusa et al. [12] to wholemount specimens of human colon (up to 100 μm thick). Using this modified protocol, 2 layers of antisera were applied to the same preparation, allowing co-localisation of 5 immunohistochemical markers in each myenteric nerve cell body [13]. In a more recent study, 12 markers were quantified in over 2500 myenteric neurons in wholemount preparations as a basis for dividing them into classes [4]. Here we report a series of 7 experiments designed to whether 2-ME/SDS works in wholemount preparations of human colonic myenteric plexus, or whether its utility is restricted to paraffin sections examined in previous published studies. Further, we aimed to characterise limitations of the method in wholemount preparations and additionally identify potential novel uses.

## 2 Materials and methods

### 2.1 Colonic tissue collection and preparation

After obtaining written informed consent for use of colonic tissue for research purposes, samples were collected from four patients undergoing elective colorectal surgery for large bowel cancer (Southern Adelaide Clinical Human Research Ethics approval number 207.17) in accordance with the Code of Ethics of the World Medical Association (Declaration of Helsinki). For the four specimens analysed, the median age was 77 years (range 71–83), three patients were female, and two specimens were from proximal colon (*n* = 1 ascending, *n* = 1 transverse) and two specimens were from distal colon (*n* = 1 descending, *n* = 1 descending/sigmoid).

A 2 cm ring of tissue was cut from the healthy margin of the colonic specimen and transported from the operating theatre to the laboratory, located in the same building, in room temperature oxygenated modified Krebs solution (NaCl; 118 mM, KCl; 4.8 mM, CaCl_2_; 2.5 mM, MgSO_4_; 1.2 mM, NaHCO_3_; 25 mM, NaH_2_PO_4_; 1.0 mM, glucose; 11 mM, bubbled with 95% O_2_, 5% CO_2_, pH 7.4). The intact ring of colonic tissue was opened up longitudinally, pinned out in a Sylgard-lined Petri dish (Sylgard 184 Elastomer, Dow, USA) serosal side down and the mucosa and submucosa were removed by sharp dissection. Tissue was re-pinned, serosa-side up in a Sylgard-lined Petri dish under maximal tension, then immersion-fixed in 4% paraformaldehyde overnight (4% paraformaldehyde in 0.1 M phosphate buffer, pH 7.2 at 4 °C). The next morning, specimens were unpinned and immersed in 4% paraformaldehyde for an additional 24 h at room temperature, with light agitation, to ensure thorough fixation.

Further dissection was performed to remove circular muscle, with care to minimise damage to the underlying myenteric plexus. Tissue was then immersed in 0.5% Triton X-100 in 0.15 M NaCl with 0.01 M phosphate buffer (PBS) pH 7.2 for two nights at room temperature to permeabilise cell membranes. The order of elution and exposure to primary, secondary, tertiary layers were varied for each of the experiments described in this study. The elution protocol and incubation times for antisera were performed as described below.

### 2.2 Elution

For the standard 2-mercaptoethanol/sodium dodecyl sulfate (2-ME/SDS) elution buffer, the solution was prepared in an extractor hood by mixing 10 mL 10% SDS, 6.25 mL 0.5 M Tris-HCl (pH 6.8), 33.75 mL distilled water and 0.4 mL 2-ME (Sigma, USA) [12]. Tissue was immersed in the 2-ME/SDS buffer, incubated in a pre-heated water bath (56 °C) for 1 h, with agitation at 60 rpm. Afterwards, the tissue was washed in PBS followed by a 2 h incubation in 0.5% Triton X-100 in PBS.

### 2.3 Primary, secondary and tertiary antisera incubations

Wholemount preparations were incubated at room temperature in primary antisera for three nights. The secondary antisera were then applied overnight and, where used, the tertiary layer (fluorophore-conjugated streptavidin) was also applied overnight. All steps were followed by three extended washes in PBS. Antisera used are summarised in Table 1.

**Table 1:** Details of primary and secondary antibodies used in the study. All secondary antisera were purchased from Jackson ImmunoResearch (West Grove, PA,USA). Abbreviations: HuCD: neuronal RNA-binding proteins C and D; nNOS: neuronal nitric oxide synthase; NF200: neurofilament-H (200kD).

Antigen	Source	Host	Code	Dilution	RRID	Secondary	Cat No.	Tertiary layer
HuC/D	Molecular Probes	mouse	A-21271	1:200	AB_221448	mouse-Biotin	715-065-151	Streptavidin AF488 Mol. Probes S-11223
nNOS	Emson	sheep	K205	1:5000	AB_2314957	sheep-AF647	713-605-147	–
NF 200	Sigma	mouse	N0142	1:1000	AB_477257	mouse- Cy3	715-165-151	–

### 2.4 Imaging

Prior to imaging, preparations were equilibrated with buffered glycerol diluted with PBS (50% -> 70% -> 100%, pH 8.6) before mounting on glass slides with a coverslip. Specimens were viewed and photographed using standard exposures on an IX71 epi-fluorescence microscope (Olympus, JPN) to record the intensity of staining or to determine if the previous layer had been effectively eluted. Some immunohistochemically labelled preparations were scanned using an Olympus VS200 slide scanner (Olympus, JPN) allowing low power, high resolution magnification of the entire specimen. Representative images in the same location as the IX71 images were captured at 20x (NA: 0.8) magnification using a LSM880 confocal microscope (Zeiss, DEU) and analysed using ImageJ software (version 2.1.0, USA). Filter sets allowed selective viewing of AMCA, FITC or Alexa Fluor® (AF) 488, Cy3 or AF555, and Cy5 or AF647.

### 2.5 Anterograde tracing

A specimen of live colon tissue was opened to make a flat sheet and pinned mucosal-side up in a sterile Sylgard-lined Petri dish. The mucosa and submucosa were removed by sharp dissection and discarded and the preparation was turned over and re-pinned. An extrinsic nerve entering the specimen was identified and dissected free of connective tissue and fat leaving a free, bare stump of a nerve trunk, approximately 5 mm long. The preparation was then cut to size (approximately 2 cm × 2 cm) and transferred to a clean, sterile Sylgard-lined Petri dish, fitted with a Perspex® chamber of 1 mL volume. The specimen was pinned next to the chamber and the nerve trunk was drawn into it through a narrow slit, which was sealed with a coverslip and silicon vacuum grease (Ajax Chemicals, AUS). The nerve trunk in the sealed chamber was rinsed with artificial intracellular solution (150 mmol/L monopotassium l-glutamic acid, 7 mmol/L MgCl_2_, 5 mmol/L glucose, 1 mmol/L ethylene glycol-bis(beta-aminoethyl ether)-N,N,N′,N′-tetraacetic acid, 20 mmol/L Hepes buffer, 5 mmol/L disodium adenosine triphosphate, 0.02% saponin, 1% dimethyl sulfoxide, 100 IU/mL penicillin, 100 μg/mL streptomycin, and 20 μg/mL gentamycin). The chamber was then filled with paraffin oil and a 20 μl drop of biotinamide solution (5% N-[2-aminoethyl] biotinamide hydrobromide, (Molecular Probes, USA) dissolved in artificial intracellular solution, was placed on the nerve trunk. In the Petri dish, Krebs solution was replaced with sterile culture medium (DME/F12 (Sigma, USA) supplemented with 10% v/v fetal bovine serum, 100 IU/mL penicillin, 100 μg/mL streptomycin, 20 μg/mL gentamycin, 2.5 μg/mL amphotericin, 1 μM nicardipine, and 1 μM hyoscine) and the dish was placed on an orbital mixer in an incubator overnight (5% CO_2_, 37 °C).

The next morning, the paraffin oil and biotinamide solution were removed from the Perspex chamber and the nerve trunk was washed repeatedly with PBS (0.1 M, pH 7.2) to remove biotinamide. The preparation was un-pinned, transferred to a new Sylgard-lined Petri dish, re-pinned under maximal tension in both circumferential and longitudinal axes and fixed as described above. The circular muscle was then removed by fine dissection and the preparation was incubated in PBS +0.5% Triton™ X-100 (PBST; X100, Sigma, USA) overnight. Nerve fibres that had taken up biotinimide were visualised by streptavidin-AF488 (incubated for 2 nights; 1:400). The tissue was then stained for human RNA binding proteins C and D (HuC/D) immunoreactivity (see above), washed 3 times in PBS, equilibrated with buffered glycerol diluted with PBS (50% -> 70% -> 100%, pH 8.6) before mounting on glass slides with a coverslip.

## 3 Results

Initially several elution buffers were compared for their ability to remove primary and fluorescently-labelled secondary antisera from wholemount preparations of human colon/myenteric plexus. These included a glycine/SDS buffer (25 mM glycine, 1% SDS, pH 2) [12], the standard 2-ME/SDS [12] and an identical buffer substituting dithiothreitol for 2-ME (DTT/SDS).

Confirming previous findings [12], incubation with the glycine/SDS buffer [14] for 1–5 h at 50 °C showed incomplete elution of antisera (*n* = 2). The DTT/SDS buffer (62 mM Tris, 2% SDS, 100 mM DTT, pH 6.75) for 1–3 h at 56 °C (*n* = 2) was more effective, however preparations showed higher background than the standard 2-ME/SDS buffer (data not shown). Incubation in 2-ME/SDS buffer [12] at 56 °C for either 1, 2 or 3 h all resulted in effective antisera removal. Therefore for all subsequent preparations we used a 1 h incubation time. Three temperatures were compared for the 2-ME/SDS buffer; 37 °C, 56 °C and 70 °C. The lowest temperature (37 °C) resulted in incomplete elution of antisera; 56 °C and 70 °C were equally effective in removing staining, however 70 °C caused greater tissue shrinkage than 56 °C (15.4 vs. 0.8% circumferentially, 14.6 vs. 10.6% longitudinally). Thus, 56 °C was selected as the optimal temperature. Similar to a previous report [12], 2-ME/SDS buffer for 1 h at 56 °C was chosen as the standard elution protocol as it was most effective for eluting staining, with the smallest effect on autofluorescence and tissue shrinkage. This was followed by rinses in PBS rather than distilled water [12].

### 3.1 General observations on the standard elution protocol

It should be noted that, in addition to effects on immunohistochemical labelling and tissue shrinkage, repeated bouts of the standard elution protocol, 2-ME/SDS, caused a small and variable increase in background fluorescence of preparations. It also strongly enhanced the autofluorescence of red blood cells throughout the specimen.

### 3.2 Experiment 1:Effect of elution on tissue epitopes

**Fig. 1:**
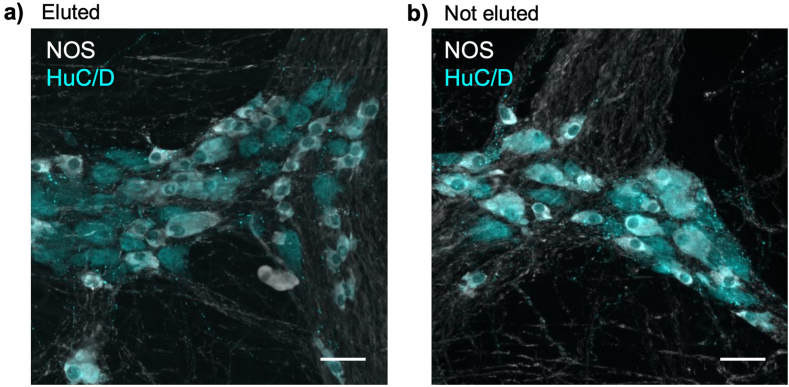
Prior exposure to 2-ME/SDS has little effect on subsequent immunohistochemical staining. Two specimens from the same patient are shown; **a)** was pre-treated with 2-ME/SDS; **b)** was not treated. Both were labelled for immunoreactivity for HuC/D (cyan) and nitric oxide synthase (NOS, white) and photographed with identical exposures. Similar intensity of labelling indicates that 2-ME/SDS does not damage tissue antigens. Scale bars = 50 μm.

Two pieces of colonic tissue were prepared from each patient. One was subjected to the standard elution protocol with exposure to 2-ME/SDS buffer: the other was not. Both were then labelled with primary antisera to HuC/D, and neuronal nitric oxide synthase (NOS) with appropriate secondary antisera. The staining of the two specimens was indistinguishable, indicating that 2-ME/SDS elution did not appear to damage epitopes in fixed tissue (Fig. 1a and b).

### 3.3 Experiment 2: elution causes shrinkage

The standard elution protocol caused measurable tissue shrinkage, especially after repeated bouts. After 3 1-h elutions, tissue shrank by 9.9 ± 4.3% circumferentially and 11.4 ± 5.9% longitudinally (*n* = 3). After 8 elutions, this increased to 14.6 ± 2.1% circumferentially and 35.5 ± 3.4% longitudinally (*n* = 3). For this reason, measurements such as cell size (from HuC/D labelling) should be made before the first elution (i.e. on the first layer of immunohistochemical labelling).

### 3.4 Experiment 3: elution removes both primary and secondary antisera

**Fig. 2:**
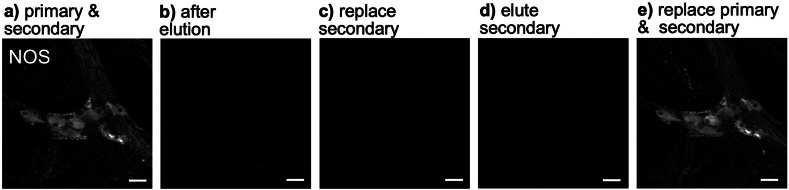
Elution removes both primary and secondary antisera from tissue. Five images of the same ganglion are shown. It was first exposed to anti-NOS primary and AF488-conjugated secondary antisera (NOS – **a)**). Tissue was then eluted **b)** which removed all NOS labelling. Incubation with the secondary antiserum alone **c)** did not restore NOS staining. After eluting the secondary antibody **d)**, incubation with primary then secondary antisera fully restored NOS labelling **e).** Scale bars = 50 μm.

Tissue was incubated with primary and secondary antisera to reveal NOS immunoreactivity (Fig. 2a). It was then eluted using the standard 2-ME/SDS protocol, which removed all visible NOS labelling (Fig. 2b). The secondary antiserum was then applied again, but did not restore NOS labelling (Fig. 2c), suggesting that the primary antibodies had also been removed or denatured by the standard elution protocol. The secondary antisera was then eluted by a second incubation in 2-ME/SDS (Fig. 2d). The tissue was then washed in PBS and re-incubated successively with both primary and secondary antisera (Fig. 2e). This fully restored NOS-labelling, confirming that the NOS tissue epitopes had not been affected by 2-ME/SDS. (Fig. 2a–e).

### 3.5 Experiment 4: staining using biotinylated antisera is resistant to elution

**Fig. 3:**
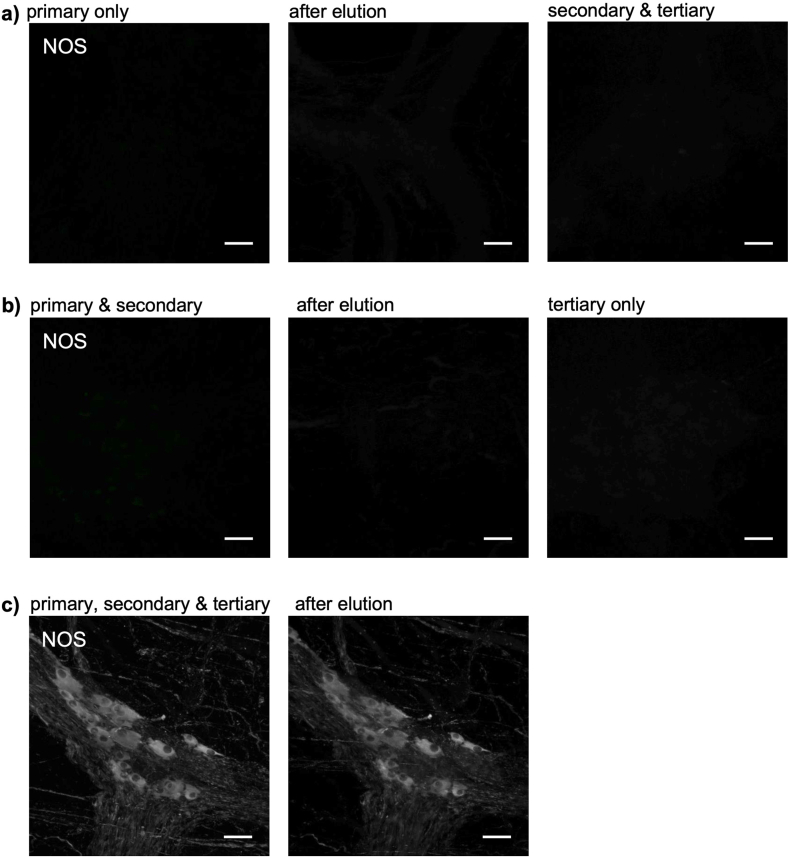
Biotin-streptavidin complexes protect labelling from elution. Three specimens from the same patient (**a, b, c**) were stained with anti-NOS primary followed by biotin-coupled secondary antiserum followed by streptavidin-AF488 tertiary. Specimen **a)** was eluted after the primary NOS antiserum, then incubated with the secondary and tertiary layers. No labelling was visible (top right), suggesting that the primary antiserum had been eluted. Specimen **b)** was exposed to the anti-NOS primary (left) followed by biotinylated secondary (left), then eluted with 2-ME/SDS (middle). Incubation with the tertiary (streptavidin-AF488) did not reveal labelling, suggesting that elution had removed both primary and biotinylated secondary from the tissue (right). In **c)**, all 3 antisera were applied in sequence, revealing intense NOS immunoreactivity (left) which was largely unaffected by subsequent 2-ME/SDS (middle). Scale bars = 50 μm.

Three specimens of colonic tissue from the same patient were stained for NOS immunoreactivity, however they underwent elution at different points in the process (Fig. 3a–c). Specimen 1 had anti-NOS primary antiserum applied, then elution, then biotinylated-secondary and tertiary (streptavidin-AF488) layers. No labelling was visible (Fig. 3a, top row), confirming that elution can remove or denature a primary antibody bound to an epitope in fixed tissue (Fig. 3a). Specimen 2 had NOS primary antisera followed by biotinylated secondary antibody, and was then subjected to elution (Fig. 3b - middle row). It was then incubated with a fluorescent tertiary (streptavidin-AF488) reagent. No labelling was visible (Fig. 3b). Specimen 3 had primary, biotinylated secondary and a tertiary layer applied leading to intense NOS labelling (Fig. 3c, bottom row). Elution was then applied after viewing and caused only a small reduction in staining intensity (Fig. 3c). This series of experiments shows that elution can remove primary antisera and biotinylated secondary antibodies, but is much less effective against the primary/secondary/tertiary combination. It appears that the formation of the biotin-streptavidin complex makes the antiserum complex more resistant to denaturation and elution. This was tested further in the next experiment.

### 3.6 Experiment 5: a biotinylated neuronal tracer is resistant to elution

**Fig. 4:**
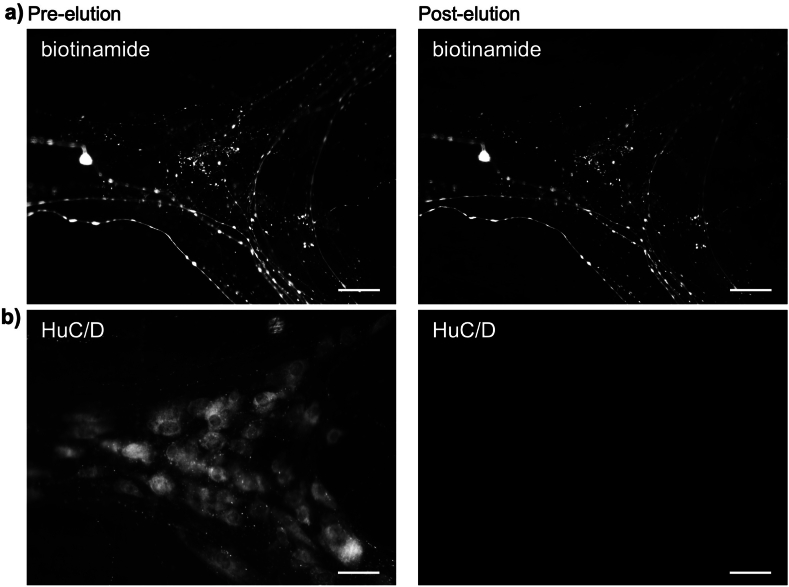
A biotinylated axonal tracer, revealed by streptavidin-AF488, was resistant to elution by 2-ME/SDS. Extrinsic axons in human colon were anterogradely labelled with biotinamide and visualised with streptavidin-AF488 (a-left). 2-ME/SDS had little effects on staining intensity (a - right). In contrast, HuC/D labelling of the same ganglion (b - left), visualised with donkey anti-mouse-AF555 secondary (left) was fully removed by the elution (right). Scale bars = 50 μm.

We tested whether a biotinylated axonal tracer (*N*-(2-aminoethyl) biotinamide hydrochloride) [15], used for rapid axonal tracing ex vivo [16] applied to extrinsic axons entering the gut wall [16] could be eluted using the standard 2-ME/SDS elution protocol. Biotinamide, applied to a human colonic nerve and revealed with streptavidin-AF488, labelled varicose branching axons within a myenteric ganglion (Fig. 4a, left side). In the same preparation, HuC/D-immunoreactivity in enteric nerve cell bodies was revealed by a HuC/D primary antiserum followed by an AF555-conjugated secondary antiserum (Fig. 4b left side). After elution, the biotinamide-labelled axons remained intensely labelled (Fig. 4a - right side) whereas the HuC/D immunoreactivity disappeared entirely (Fig. 4b - right side). This suggests that the biotin-streptavidin complex confers resistance to elution and that this does not require the biotin to be conjugated to a secondary antiserum.

### 3.7 Experiment 6: labelling with two primary antisera raised in the same species

**Fig. 5:**
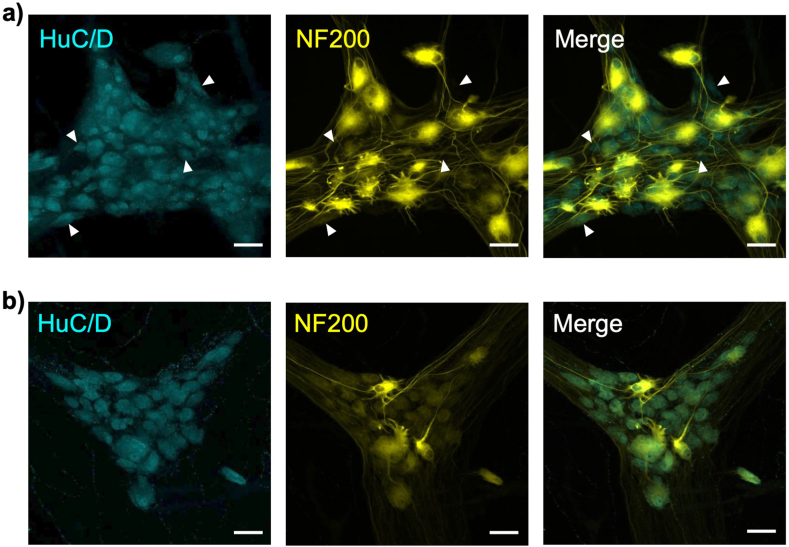
Labelling with two primary antisera raised in the same species. Two specimens from the same patient were both incubated with primary antiserum raised in mouse to HuC/D, biotinylated secondary and a streptavidin-AF488, revealing immunoreactive myenteric nerve cell bodies (cyan - left column). Specimen **a)** was then exposed to 2-ME/SDS; while specimen **b)** was kept in PBS. Both specimens were then incubated with a primary antiserum to NF200 (yellow) also raised in mouse, followed by secondary conjugated to AF555 (middle). In **b)**, every HuC/D-immunoreactive cell body was also labelled in AF555. However in **a)**, some HuC/D-positive cell bodies lacked AF555 labelling entirely (white arrowheads). The 2-ME/SDS may have denatured epitopes of the primary anti-HuC/D and biotinylated secondary so that the NF200 primary and AF555-secondary no longer bound. Scale bars = 50 μm.

Two specimens of colonic tissue from the same patient were incubated with a HuC/D primary antiserum raised in mouse. This was followed by a biotinylated donkey-*anti*-mouse secondary and a fluorescent tertiary layer (streptavidin-AF488). This gave rise to intense HuC/D labelling in both preparations. One specimen was then subjected to the standard 2-ME/SDS elution protocol followed by PBS washes, while the other only had the PBS washes. Both specimens were then incubated with an antiserum to a primary neurofilament-H (NF200) antiserum also raised in mouse. This was followed by a donkey-*anti*-mouse-AF555 secondary antiserum. In the tissue that had been exposed to the standard elution protocol (Fig. 5a - top row), many HuC/D cells lacked immunoreactivity for NF200 (white arrowheads), whereas in tissue that was not eluted (Fig. 5b - bottom row) all HuC/D cells were labelled for NF200 (Fig. 5). This suggests that the elution step prevented cross-reactivity between the second layer of primary and secondary antisera (for NF200) and the antisera in the first layer (for HuC/D).

### 3.8 Experiment 7: elution reduces lipofuscin autofluorescence

**Fig. 6:**
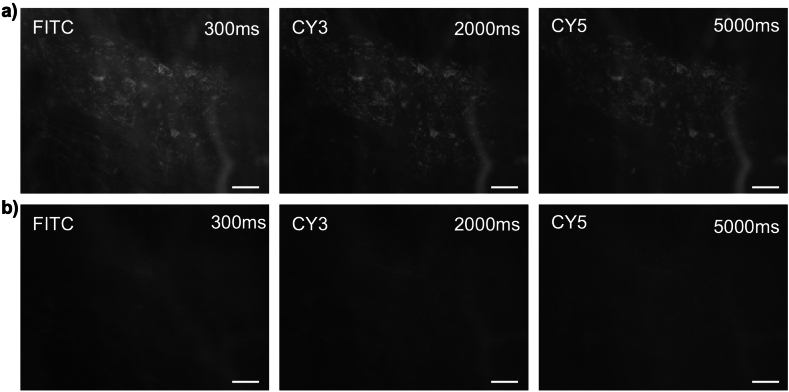
Lipofuscin autofluorescence is reduced after elution. Prior to elution, lipofuscin was visible in all 3 filters **a)**. After elution **b)** it is largely absent. Images were captured on an IX71 epifluorescence microscope with matched exposures (FITC: 300 ms, CY3: 2s and CY5: 5s). Scale bars = 50 μm.

In experiment 7, four specimens of colonic tissue from four patients were imaged before exposure to antisera. Filters for FITC, CY3 and CY5 all revealed dim, granular autofluorescence of lipofuscin in all 4 specimens. Micrographs were taken with timed exposures (Fig. 6a - top row). Each preparation was then eluted using the standard protocol and re-imaged using the same timed exposure. Three of the four specimens showed a significant reduction in lipofuscin autofluorescence after elution (Fig. 6b - lower row); in one patient's tissue the reduction was less marked.

### 3.9 Evaluation of cost

The costs associated with use of 2-ME/SDS are modest. The main ingredients are 2-mercaptoethanol (2-ME), sodium dodecyl sulfate (SDS), Tris-HCl (pH 6.8), and distilled water, none of which is expensive. Total cost of ingredients in 50 mls of 2-ME/SDS (enough for 4 or more preparations) is approximately USD $5.00. No special equipment is required apart from a temperature-controlled waterbath with agitation.

## 4 Discussion

The development of fluorescent-labelled antibodies [17] made immunohistochemical localisation of antigens in cells and tissues possible, with detected antigens ranging from small molecules to large proteins. Subsequently, multiple-labelling became possible by having different cellular markers coupled to different fluorophores. This led to the demonstration of co-existence of neurochemical markers in single nerve cells [18]; a major advance for neuroscience. Within a few years it was realised that different classes of neurons could be distinguished by the combinations of markers that they express; sometimes referred to as “chemical coding" [19]. Since then, immunohistochemical characterisation has been widely used in many parts of the nervous system to distinguish different types of neurons. However, multiple-labelling immunofluorescence histochemistry is typically restricted to 3-4 markers, limiting the utility of such studies. The ability to elute antisera and re-stain preparations provides a straightforward means to overcome this restriction. Importantly, this method works with commercially-available primary/secondary antibody combinations and does not require the libraries of directly-labelled primary antibodies used in some other methods [9,10]. The standard elution protocol used in the present study was originally used in 3–4 μm thick sections of paraffin-embedded tissue [12]. The solution had been derived from the stripping buffers that are routinely applied to nitrocellulose or polyvinylidene difluoride membranes for re-probing western blots with multiple primary antibodies. The present work and our recent studies [4],[13,20] showed that this technique also works effectively in wholemount preparations of human colon. In our hands, the method has been tested with up to 9 layers of labelling of wholemount preparations of human gut tissue. The main limit to the number of elutions was the damage to the tissue caused during repetitive dismounting and remounting and the accumulation of detritus during exposure to multiple solutions.

### 4.1 Likely mechanism of action

2-mercaptoethanol is a potent reducing agent that breaks disulphide crosslinks within proteins and SDS denatures the tertiary structure of proteins, which disrupts many epitopes. However, 2-ME/SDS did not appear to damage epitopes in fixed tissue (experiment 1), suggesting that the cross-linking by formaldehyde during fixation protects proteins from denaturation. In contrast, the 2-ME/SDS buffer effectively removed both primary and secondary antibodies bound to tissue. The antisera used for immunofluorescence were not exposed to formaldehyde fixation and hence are labile proteins, which may explain their susceptibility to denaturing by 2-ME/SDS, while epitopes in the fixed tissue were unaffected.

It was previously reported that labelling by biotinylated secondary antisera followed by a streptavidin-conjugated fluorophore “tertiary" was resistant to elution [12]; this result was confirmed in experiment 4 of the present study. Furthermore, it was shown that biotinamide; a fixable modified biotin analogue used as a neural tracer, was similarly resistant to elution with 2-ME/SDS when bound to a streptavidin-conjugated fluorophore (experiment 5). Streptavidin binds biotin with an extremely strong covalent bond with an equilibrium dissociation constant of 4 × 10^−14^ M. In effect, the tightly bound 60kD streptavidin protein may protect underlying proteins from degradation. Streptavidin lacks disulphide crosslinks [12], which are a major site of action of 2-ME. These two factors may explain why biotin-streptavidin complexes are resistant to elution, whether they are linked to secondary antisera or as a stand-alone neuronal tracer. In contrast, bare IgG molecules used as primary or secondary antisera in this study classically have 12 disulphide bonds, explaining their sensitivity to the 2-ME/SDS buffer. This resistance to elution was exploited in a recent study in which all enteric nerve cell bodies were labelled with the pan-neuronal marker HuCD, visualised with biotinylated secondary and AMCA-coupled streptavidin. The cell bodies could then be individually identified in 5 subsequent layers of labelling in which 12 selective cell body markers were applied, providing a detailed chemical coding profile of each neuron [4].

Selective denaturation can be used to label tissue with two primary antisera raised in the same host species (experiment 6). For this to work, the order of application of reagents was important. The first primary had to be immediately followed by a biotinylated secondary and tertiary streptavidin-fluorophore. The tissue was then exposed to 2-ME/SDS followed by the second primary antibody, succeeded by the second fluorescent secondary antiserum. We speculate that the 2-ME/SDS step disrupts the unused antigen-recognition site on the first secondary, preventing them from subsequently binding the second primary. Similarly, 2-ME/SDS denatures exposed epitopes on the first primary that would normally cross-react with the second secondary antiserum which is applied in the second layer of staining.

### 4.2 Lipofuscin autofluorescence

The ability of 2-ME/SDS treatment to reduce lipofuscin autofluorescence was not anticipated. Lipofuscin is a granular, heterogenous mixture of oxidised protein, lipids and lipoproteins with small amounts of metal ions (iron, copper, zinc) that accumulates with age in the cytoplasm of post-mitotic cells, including neurons [21]. Lipofuscin typically autofluoresces across a broad range of visible wavelengths and can interfere with immunofluorescence signals. The ability of the 2-ME/SDS solution to reduce lipofuscin autofluorescence was in stark contrast to its lack of effect on epitope binding in fixed preparations (experiment 1), suggesting that the quenching may not be solely due to denaturation of lipofuscin-associated proteins. Sudan Black B, a lipophilic dye has previously been used to quench lipofuscin autofluorescence in cells, with minor effects on immunofluorescence signal in CNS tissues [22] and the enteric nervous system [23]. Since 2-ME/SDS elution reduces lipofuscin autofluorescence it may remove the need to perform extra steps such as staining with Sudan Black B during multi-labelling immunohistochemistry.

### 4.3 Uses of antibody elution

A major use of the antibody elution technique is to increase the numbers of markers that can be assayed in cells within a specimen. This requires that the same cells can be identified reliably between different layers of staining. In wholemount preparations of enteric plexuses this can be achieved by the application of HuC/D antisera, which label nearly all enteric nerve cell bodies [13,24]. Individual cell bodies can then be reliably re-identified by size, shape and location in ganglia, relative to other cells [4]. The use of biotinylated secondary antiserum to reveal HuC/D can facilitate this process by making HuC/D labelling resistant to elution so that it persists between layers of antisera without requiring re-staining. For neuronal tracing studies, biotinylated axonal markers are similarly resistant to elution by the standard protocol, so that sparsely labelled single axons can be re-identified between layers of antisera (data not shown).

The ability to elute antisera has several other uses. Some specimens of human tissue, including pathological samples, are very rare and hence valuable. Wholemount human tissue specimens can require painstaking dissection and this further increases their value. Thus, the ability to stain such preparations more than once ensures that researchers can maximise data generated from such specimens. Furthermore, in any laboratory, poor labelling sometimes occurs due to incorrect antibody dilutions, or other mistakes. On these occasions, being able to elute antibodies gives a “second chance" to stain tissue correctly.

### 4.4 Limitations and future directions

Several previous studies used 2-ME/SDS to elute antibodies for multiplexed immunohistochemical studies of sections of formaldehyde-fixed paraffin-embedded tissue, after antigen retrieval [12,25,26]. The present study was entirely carried out on fixed, dissected wholemounts of human colonic myenteric plexus which are typically 100–200 μm thick; this makes it impossible to quantify small changes in autofluorescence or background labelling. The study of neurons in thick preparations gives important information about the soma-dendritic morphology of cells and their locations which can be correlated with the combinations of immunohistochemical markers that they express [4]. The present study used a restricted range of antisera which may not be entirely representative; studies in sectioned material have reported variations in the efficacy of 2-ME/SDS in larger samples of primary/secondary antibody combinations [12,25]. The value of multiplexed immunohistochemical labelling lies in being able to identify distinctive combinations of markers in individual cells. Fiducial markers were not required to align images from different layers of staining [9] because in wholemounts of human myenteric plexus nerve cell bodies are sparsely distributed and can be uniquely identified by their morphology and location in ganglia [4]. However, tissue shrinkage and small variations in orientation of restained preparations meant that co-existence of markers across layers could not be reliably determined at the sub-cellular level (in axonal varicosities).

All steps in this study were performed manually on isolated specimens of tissue; this is very time-consuming, taking up to 30 days to process a single tissue sample through 5 layers [4]. Identifying ways to automate the processing would be invaluable in the future. The ability to incorporate fine-scale fiducial points to allow automatic alignment of images at the level of individual axons would be a powerful addition. Future studies could relate immunohistochemical co-existence of markers to molecular analysis at the single cell level using RNA-seq methodology [27].

## 5 Conclusions

The use of 2-ME/SDS solution to elute antisera was introduced as part of a method for multiplexed immunohistochemical labelling of thin sections of formaldehyde-fixed human pathological tissue [12]. The current study has demonstrated similar efficacy in wholemount preparations, for multiplexed analysis of nerve cell bodies and shown that it has minimal effects on epitopes in fixed tissue. Biotin-streptavidin binding (in either antisera or a non-antibody based neuronal tracer) was confirmed to confer resistance to elution by 2-ME/SDS. These characteristics led to identification of a new protocol for simultaneous labelling with 2 primary antisera raised in the same species. In addition, shrinkage caused by 2-ME/SDS was quantified and its ability to reduce lipofuscin fluorescence was noted. This low cost method is worth exploring when considering immunohistochemical analysis of nerve cell bodies in wholemount specimens.

## Funding

This work was supported by 10.13039/100000002National Institutes of Health (NIH)- Stimulating Peripheral Activity to Relieve Conditions (SPARC) award OT2OD24899 to Y Taché, 10.13039/100005916UCLA.

## Data availability

Data used in this study will be made available via the publicly accessible data repository Pennsieve with https://doi.org/10.26275/k11r-zl51.

## Additional information

No additional information is available for this paper.

Table 1. Details of primary and secondary antibodies used in the study. All secondary antisera were purchased from Jackson ImmunoResearch (West Grove, PA, USA). Abbreviations: HuCD: neuronal RNA-binding proteins C and D; nNOS: neuronal nitric oxide synthase; NF200: neurofilament-H (200kD).

## CRediT authorship contribution statement

**Adam Humenick:** Writing – review & editing, Writing – original draft, Investigation, Formal analysis, Data curation, Conceptualization. **M.E. Johnson:** Writing – review & editing, Writing – original draft, Investigation, Formal analysis, Data curation, Conceptualization. **B.N. Chen:** Writing – review & editing, Writing – original draft, Investigation, Formal analysis, Data curation, Conceptualization. **M. Wee:** Writing – review & editing, Writing – original draft, Investigation, Data curation. **D.A. Wattchow:** Writing – review & editing, Writing – original draft, Funding acquisition, Conceptualization. **M. Costa:** Writing – review & editing, Writing – original draft, Investigation, Formal analysis, Conceptualization. **P.G. Dinning:** Writing – review & editing, Writing – original draft, Funding acquisition, Data curation, Conceptualization. **S.J.H. Brookes:** Writing – review & editing, Writing – original draft, Supervision, Resources, Project administration, Methodology, Investigation, Funding acquisition, Formal analysis, Data curation, Conceptualization.

## Declaration of competing interest

The authors declare that they have no known competing financial interests or personal relationships that could have appeared to influence the work reported in this paper.
